# Growth differentiation factor-15 is associated with cardiovascular outcomes in patients with coronary artery disease

**DOI:** 10.1186/s12933-020-01092-7

**Published:** 2020-08-03

**Authors:** Man Li, Lei Duan, Yu-Lun Cai, Hui-Ying Li, Ben-Chuan Hao, Jian-Qiao Chen, Hong-Bin Liu

**Affiliations:** 1 Medical School of Chinese PLA, Beijing, China; 2 Department of Cardiology, The Second Medical Center, Chinese PLA General Hospital, Beijing, China; 3 Beijing Key Laboratory of Chronic Heart Failure Precision Medicine, Beijing, China

**Keywords:** Growth differentiation factor-15, Major adverse cardiovascular outcomes, Coronary artery disease

## Abstract

**Background:**

Growth differentiation factor-15 (GDF-15) is a marker of inflammation, oxidative stress and it is associated with adverse prognosis in cardiovascular disease. The aim of the present cohort study is to investigate the prognostic value of GDF-15 in patients with coronary artery disease (CAD) during long-term follow up.

**Methods:**

A total of 3641 consecutive patients with CAD were prospectively enrolled into the study and followed up for major adverse cardiovascular events (MACEs) and all-cause death up to 5.3–7.6 years. Plasma GDF-15 was measured and clinical data and long-term events were registered. The patients were subsequently divided into three groups by the levels of GDF-15 and the prognostic value of GDF-15 level with MACEs and all-cause death was evaluated.

**Results:**

After a median follow-up at 6.4 years later, 775 patients (event rate of 21%) had developed MACEs and 275 patients died (event rate of 7.55%). Kaplan–Meier analysis indicated that the patients with GDF-15 > 1800 ng/L were significantly associated with an increased risk of MACEs and all-cause death. Cox regression analysis indicated that GDF-15 > 1800 ng/L were independently associated with the composite of MACEs (HR 1.74; 95% CI 1.44–2.02; P < 0.001) and all-cause death (HR 2.04; 95% CI 1.57–2.61; P < 0.001). For MACEs, GDF-15 significantly improved the C-statistic (area under the curve, 0.583 [95% CI 0.559–0.606] to 0.628 [0.605–0.651]; P < 0.001), net reclassification index (0.578; P = 0.031), and integrated discrimination index (0.021; P = 0.027). For all-cause death, GDF-15 significantly improved the C-statistic (0.728 [95% CI 0.694–0.761] to 0.817 [0.781–0.846]; P < 0.001), net reclassification index (0.629; P = 0.001), and integrated discrimination index (0.035; P = 0.002).

**Conclusions:**

In the setting of CAD, GDF-15 is associated with long-term MACEs and all-cause death, and provides incremental prognostic value beyond traditional risks factors.

## Background

Cardiovascular (CV) diseases, consisting of ischemic heart disease, stroke, heart failure, peripheral arterial disease, and a number of other cardiac and vascular conditions, remains the leading cause of death of the world [1]. It is well known that coronary heart disease as a main cause of ischemic heart disease has become a major health concern over the past several decades. Patients with previous coronary heart disease have a high probability of MACEs. Stratification for subsequent coronary events among patients with coronary artery disease (CAD) is of considerable importance because of the potential to guide secondary preventive therapies. Identifying high-risk patients prone to future MACEs may improve prognosis. Conventional risk factors that include gender, age, smoking, glucose level, blood pressure and cholesterol level have long been used to risk stratify subjects who are at risk of MACEs [2, 3]. However, these clinical risk factors themselves have limited predictive value in patients with CAD. Biomarkers have become increasingly important tools helping to improve patient care over the past two decades. In recent years, cardiac biomarkers have been shown to be increasingly important tools helping to predict cardiovascular risk and superior to models based solely on traditional risk factors [4, 5]. Growth and differentiation factor 15 (GDF-15), previously known as macrophage inhibitory cytokine 1 (MIC-1), is a divergent transforming growth factor β (TGF-β) family member historically associated with cancer cachexia, cardiovascular disease, and a host of other diseases with inflammatory etiologies [6]. GDF-15 is expressed in most tissues only at very low levels [7], the only human organ that expresses high levels of GDF-15 in healthy conditions is the placenta [8]. However, GDF-15 could be markedly increased in the case of cardiovascular injury, such as pressure overload, myocardial infarction, heart failure, and atherosclerosis [9–11]. In the past decade, accumulating evidence has demonstrated that the GDF-15 serve as a potential prognostic factor in patients in with acute coronary syndrome [12–14]. However, these studies did not elucidate the long-term prognostic value of MACEs in CAD patients. Therefore, the aim of the present study was to investigate the long-term prognostic value of plasma GDF-15 on all-cause death and MACEs in a large scale patients during a long-term follow up with established CAD.

## Methods

### Study population

**Fig. 1:**
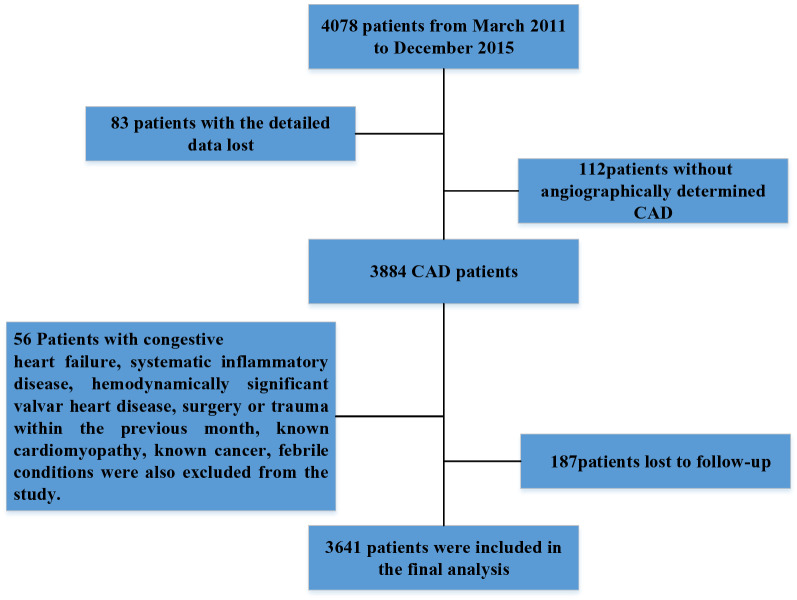
Flowchart of the study

The present study was designed as a single-center, observational cohort study. As described in the flowchart (Fig. 1), from March 2011 to December 2015, 4078 patients who underwent coronary angiography at the Chinese PLA General Hospital were recruited in the study. The patients underwent coronary angiography examination because of angina-like chest pain or positive noninvasive tests (such as treadmill exercise test or coronary computed tomography angiography). The result of angiography suggested at least one major coronary artery stenosis ≥ 50% was diagnosed as CAD. Then, 83 patients with the detailed data lost and 112 patients without angiographically determined CAD were excluded. 56 patients with congestive heart failure, systematic inflammatory disease, hemodynamically significant valvar heart disease, surgery or trauma within the previous month, known cardiomyopathy, known cancer, febrile conditions were also excluded from the study. 187 patients lost to follow-up were also excluded. Thus, the final study cohort consisted of 3641 patients, 2742 with symptoms of unstable angina or myocardial infarction (MI) and 899 with symptoms of stable angina. All subjects gave written informed consent. This study was approved by the Ethics Board of the Chinese PLA General Hospital and written informed consent was obtained from each patient.

### Baseline examinations

All enrolled patients were required to complete a standardized questionnaire to collect comprehensive data on medical and family history, medication use, smoking status, body weight, height, systolic and diastolic blood pressure. Hypertension was defined as blood pressure ≥ 140/90 mm Hg or receiving antihypertensive treatment. Hyperlipidemia was defined as known but untreated dyslipidemia or current treatment with lipid-lowering medications. Diabetes mellitus was defined as known untreated diabetes and/or use of insulin or oral hypoglycemic agents. Patients were defined as current smoker if they reported any tobacco use in the last 30 days.

### Laboratory analyses

Blood samples were drawn from patients early in the morning after hospital admission. Serum was centrifuged within 30 min, and plasma was stored at − 80 °C for subsequent analysis. Concentration of GDF-15 was routinely measured by an established available enzyme linked immunosorbent assay kit (pre-commercial Elecsys^®^ assay, measuring range 400–40,000 ng/L; Roche Diagnostics, GmbH, Mannheim, Germany). The detection limit was 400 ng/L, and the intra and inter-assay imprecisions were < 0.9% and < 2.3%, respectively. All GDF-15 measurements were performed by investigators that were not aware of patients’ characteristics and outcomes. As previously reported, GDF-15 risk categories were defined as low risk (< 1200 ng/L), intermediate (1200–1800 ng/L) and high risk (> 1800 ng/L) [12–15]. Other routine measurements were performed at the participating study centers using standard laboratory techniques.

### Follow up and study endpoints

All data were prospectively collected and entered into a database. All patients were followed up semiannually through telephone interviews or clinic visits. The primary endpoint was the occurrence of MACEs. MACEs was determined as a composite of all-cause mortality, nonfatal such as acute coronary syndrome (ACS), or unplanned revascularization treatment. All deaths were considered cardiac unless a definitive non cardiac cause was established. ACS was defined as the clinical diagnosis of ST-segment elevation myocardial infarction (STEMI), non STEMI, or unstable angina pectoris in accordance with the guidelines of the European Society of Cardiology. Unplanned coronary revascularization was defined as unplanned repeated percutaneous coronary intervention (PCI) or unplanned coronary artery bypass grafting (CABG), with at least 1 of the following: (1) recurrence of angina; (2) positive noninvasive test; and (3) positive invasive physiological test. The second outcome of this study was all-cause death.

### Statistical analysis

Continuous variables were presented as means ± SDs, while categorical variables were described as percentages. Comparisons between groups were performed by using unpaired Student’s t test for continuous variables and the Chi square test or Fisher’s exact test for categorical variables. Spearman’s correlation coefficients were calculated to evaluate the relations between the levels of GDF-15 and clinical variables. Multiple linear regression was used to determine which covariates were independently associated with GDF-15 levels. For patients who experienced more than one event, the first was considered. Kaplan–Meier analysis was used for stratified analysis of time-to-event for 2 event types: (1) MACEs; (2) overall mortality; and statistical assessment was performed using the log-rank test. The differences in proportions in outcome events in the different strata of GDF-15 levels were judged by Fisher’s exact test. The relation of GDF-15 levels to each clinical outcome is presented as cumulative Kaplan–Meier curves and analyzed with Cox proportional hazards models [hazard ratios (HRs) with 95% CIs] with GDF-15 both with GDF-15 concentration as a continuous variable and with GDF-15 group (G1-G3) as a categorical variable. Simple Cox-regression analysis was used to identify predictors of each clinical outcome during the follow up. A multivariate Cox proportional hazards regression model was used to identify independent predictors of clinical events. Covariates that were either statistically significant on univariate analysis or clinical risk factors were considered candidate variables. Model 1: Clinical background characteristics included age, sex, BMI, smoking, hypertension, diabetes mellitus, and hyperlipidemia. Model 2 includes model 1, with the addition of GDF-15. For illustrative purposes, receiver operating characteristics (ROC) plots were derived from univariable binary logistic regression models. The prognostic discrimination of GDF-15 was assessed by comparing the incremental improvement of the Brier score, Harrell’s C-statistic, and by determining the integrated discrimination improvement (IDI) and the net re-classification improvement (NRI) using continuous NRI and NRI at the event rate. All probability values were 2-sided, and P values < 0.05 were considered statistically significant. STATA 14.2 (Stata Corp, College Station, Texas, USA) and R statistical software version 3.4.4 (R Foundation for Statistical Computing, Vienna, Austria) were used for statistical analysis.

## Results

### Patient characteristics

A total of 3641 patients diagnosed as CAD admitted to our hospital were enrolled in the present study. The median age was 64 years; 56.1% were male, the median GDF-15 level was 1884 ng/L. The enrolled patients were divided into three groups upon the levels of serum GDF-15 (G1: GDF-15 < 1200 ng/L, G2: GDF-15:1200–1800 ng/L, G3: GDF-15 > 1800 ng/L). During 6.4 (median follow-up of 6.4 [interquartile range 5.3–7.6]) years of follow-up, 775 patients had an occurrence of MACEs. In those patients, 158 (15.9%) had values of GDF-15 below 1200 ng/L, 134 (17.8%) between 1200 and 1800 ng/L and 483 (25.2%) above 1800 ng/L. The baseline characteristics of the three groups were shown in Table 1.

**Table 1:** Baseline clinical and laboratory characteristics of the study patients according to status of GDF-15

	Total n = 3641	Low GDF-15 (< 1200 ng/L) (n = 991)	Medium GDF-15 (1200–1800 ng/L) (n = 750)	High GDF-15 (> 1800 ng/L) (n = 1900)	P value for trend
Age, years	61.4 (27–95)	57.2 (26–95)	59.9 (30–92)	64.3 (27–95)	< 0.001
Male, n (%)	2632 (72.29)	735 (74.17)	540 (53.33)	1357 (71.42)	0.484
BMI (kg/m2)	25.64 (13.3–41)	26.02 (13.3–41)	25.82 (16.5–37.4)	25.38 (17.5–32.1)	0.074
Current smokers, n (%)	1668 (45.82)	473 (47.72)	345 (46.00)	850 (44.70)	0.304
Hypertension, n (%)	2370 (65.09)	592 (59.74)	474 (63.20)	1304 (68.63)	< 0.001
Hyperlipidemia, n (%)	1120 (30.76)	282 (28.5%)	230 (30.70)	608 (32.0)	0.034
Diabetes mellitus, n (%)	1163 (31.94)	223 (22.50)	226 (30.13)	714 (37.58)	< 0.001
Previous MI, n (%)	254 (6.98)	54 (5.45)	44 (5.89)	156 (8.21)	< 0.001
Previous PCI/CABG, n (%)	299 (8.21)	66 (6.66)	57 (7.58)	176 (9.26)	< 0.001
TC (mmol/L)	4.03 ± 1.0	3.97 ± 1.02	4.03 ± 1.09	4.10 ± 1.14	0.046
HDL-C (mmol/L)	1.07 ± 0.68	1.06 ± 0.43	1.09 ± 0.94	1.07 ± 0.67	0.624
LDL-C (mmol/L)	2.40 ± 0.91	2.36 ± 0.84	2.44 ± 0.90	2.40 ± 0.96	0.201
TG (mmol/L)	1.62 ± 1.21	1.65 ± 1.11	1.66 ± 1.05	1.60 ± 1.32	0.326
Medications					
Aspirin, n (%)	3415 (93.79%)	942 (95.06%)	718 (95.73%)	1755 (92.37%)	0.067
ACEI, n (%)	1503 (41.28%)	403 (40.67%)	305 (40.67%)	822 (43.26%)	0.289
β-blocker, n (%)	1629 (44.74%)	744 (75.08%)	533 (71.07%)	1352 (71.16%)	0.070
Statins, n (%)	3442 (94.53%)	944 (95.25%)	716 (95.47%)	1782 (93.79%)	0.070

Patients with a higher level of GDF-15 were older, had a higher level of total cholesterol (TC), had a history of myocardial infarction and percutaneous coronary intervention or coronary artery bypass graft; and had a higher rate of hypertension, hyperlipidemia, and diabetes. There were no differences between patients included and not included in the analysis regarding other background variables: sex, body mass index (BMI), triglyceride (TG), high density lipoprotein cholesterol (HDL-C), low density lipoprotein cholesterol (LDL-C), smoking and treatment.

### Correlations of serum GDF-15 levels with other clinical biochemical factors

Increasing levels of GDF-15 at presentation were associated with increased age, diabetes, hypertension, hyperlipidemia and a history of previous myocardial infarction and previous PCI/CABG. GDF-15 levels were also related to a lower rate of aspirin use (Table 2). By multiple regression analysis that included all patients’ characteristics shown in Table 2, used the natural logarithm of GDF-15 as the dependent variable, GDF-15 was independently associated with age (P < 0.001), diabetes (P < 0.001).

**Table 2:** Spearman’s correlation coefficients between GDF-15 and clinical and biochemical parameters

	Spearman correlation	
	Coefficient	P
Age, years	0.267	< 0.001
Male, n (%)	− 0.025	0.133
BMI (kg/m^2^)	− 0.024	0.100
Current smokers, n (%)	− 0.025	0.127
Hypertension, n (%)	0.079	< 0.001
Hyperlipidemia, n (%)	0.053	0.031
Diabetes mellitus, n (%)	0.137	< 0.001
Previous MI, n (%)	0.064	< 0.001
Previous PCI/CABG, n (%)	0.096	< 0.001
TC (mmol/L)	0.017	0.315
HDL-C (mmol/L)	0.007	0.656
LDL-C (mmol/L)	0.014	0.403
TG (mmol/L)	− 0.022	0.192
Medications		
Aspirin, n (%)	− 0.057	0.001
ACEI, n (%)	0.025	0.134
β-blocker, n (%)	− 0.031	0.050
Statins, n (%)	− 0.032	0.055

### Clinical outcomes

#### Primary endpoint

A composite of major adverse cardiovascular events was analyzed during follow-up (Fig. 1).

**Fig. 2:**
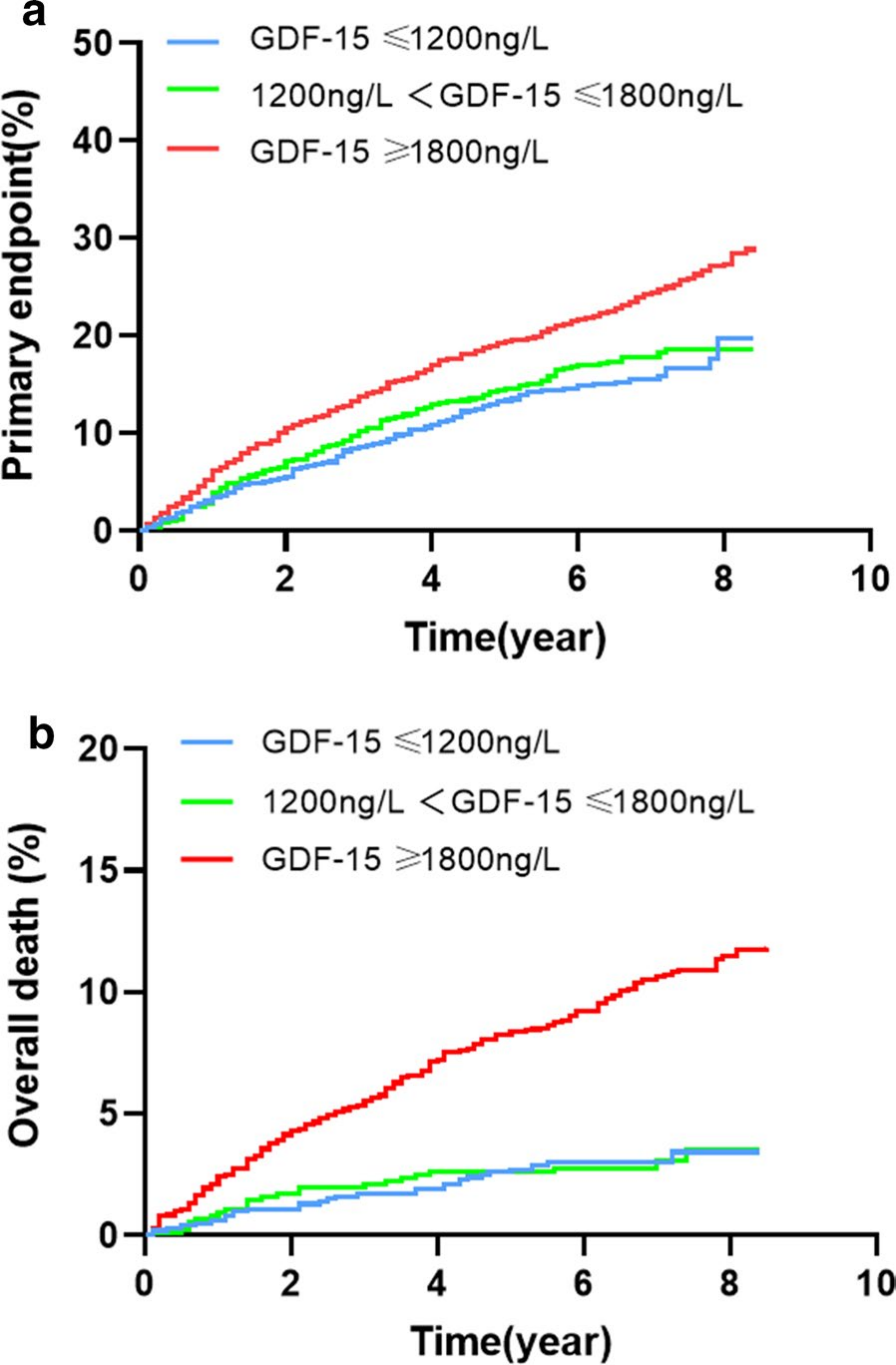
Kaplan–Meier analysis according to different levels of GDF-15

In this way, 775 patients had an occurrence of MACEs. Of those patients, 158 (15.9%) had values of GDF-15 below 1200 ng/L, 134 (17.8%) between 1200 and 1800 ng/L and 483 (25.2%) above 1800 ng/L. The MACEs rate was significantly higher in the group of patients with GDF-15 values > 1800 ng/L compared with those with GDF-15 levels between 1200 and 1800 ng/L and patients with GDF-15 values < 1200 ng/L (25.2% vs 17.8% vs 15.9%, P < 0.001). Kaplan–Meier curve of the incidence of the primary endpoint is presented in Fig. 2a. The incidence of the primary endpoint in the G3 group was significantly higher than that in the G1, G2 group (log-rank P < 0.001). Univariate Cox proportional analyses revealed that GDF-15 values > 1800 ng/L were significantly associated with the incidence of MACEs (unadjusted HR = 1.92; 95% CI 1.37–2.52; P < 0.001). After adjusted for basic clinical risk factors (age, sex, smoking hypertension, body mass index, diabetes mellitus and hyperlipidemia), in multivariate analysis, GDF-15 values > 1800 ng/L was associated with the incidence of MACEs with an HR of 1.74 (95% CI 1.44–2.02; P < 0.001) (Table 3).

**Table 3:** Relation of the GDF-15 level and MACEs in univariate and multivariate survival analysis

	Independent predictors of major adverse cardiac events					
	Univariate models			Multivariate models		
	HR	95% CI	P	HR	95% CI	P
Age	1.02	1.013−1.026	0.00	1.02	1.009−1.022	0.00
Sex	0.00	0.001−0.028	0.00	0.01	0.001−0.057	0.00
Smoking	1.10	0.933−1.296	0.23	–	–	–
BMI	0.98	0.959−1.000	0.05	1.00	0.977−1.020	0.89
Hypertension	1.03	0.646−0.880	0.00	0.82	0.703−0.967	0.02
Hyperlipidemia	1.15	0.98–1.34	0.09	–	–	–
DM	0.79	0.680–0.911	0.00	0.89	0.767–1.032	0.14
GDF-15 ≤ 1200 ng/L	1.05	0.87–1.44	0.16	–	–	–
1200 < GDF-15 ≤ 1800 ng/L	1.09	0.90–1.65	0.08	–	–	–
GDF-15 > 1800 ng/L	1.92	1.37–2.52	< 0.001	1.74	1.44–2.02	< 0.001

### Secondary endpoint

During 6.4 years of follow-up (median follow-up of 6.4 [interquartile range 5.3–7.6] years), 275 patients died. Patients with GDF-15 levels < 1200 ng/L had a low mortality rate of 3.2% (32). Patients with GDF-15 levels between 1200 and 1800 ng/L had the same mortality rate of 3.2% (24), whereas those with GDF-15 levels > 1800 ng/L had a very high mortality rate of 11.3% (219) (P < 0.001). Kaplan–Meier curve of the incidence of the primary endpoint is presented in Fig. 2b. The incidence of all-cause death the G3 group was significantly higher than that in the G1, G2 group (P log-rank < 0.001). Univariate Cox proportional analyses revealed that GDF-15 > 1800 ng/L were significantly associated with the incidence of all-cause death (Table 3). After adjustment for potential confounders, in multivariate analysis, higher GDF-15 values > 1800 ng/L were still independently associated with all-cause death (adjusted HR 2.04; 95% CI 1.57–2.61; P < 0.001) (Table 4).

**Table 4:** Relation of the GDF-15 level and all-cause death in univariate and multivariate survival analysis

	Independent predictors of all-cause death					
	Univariate Models			Multivariate Models		
	HR	95% CI	P	HR	95% CI	P
Age	1.97	1.04–1.12	< 0.001	1.07	1.01–1.09	0.01
Sex	1.23	0.98–1.37	0.09	–	–	–
Smoking	0.87	0.80–1.17	0.39	–	–	–
BMI	0.99	0.95–1.02	0.48	–	–	–
Hypertension	0.98	0.74–1.28	0.87	–	–	–
Hyperlipidemia	1.54	1.38–1.76	0.02	1.48	1.38–1.86	0.03
DM	1.11	0.87–1.42	0.42	–	–	–
GDF-15 ≤ 1200 ng/L	1.11	0.87–1.42	0.42	–	–	–
GDF-15 ≤ 1800 ng/L	1.33	0.94–1.56	0.13	–	–	–
GDF-15 > 1800 ng/L	2.54	1.99–3.09	< 0.001	2.04	1.57–2.61	< 0.001

### Incremental value of GDF-15 over conventional risk factors for MACEs

**Fig. 3:**
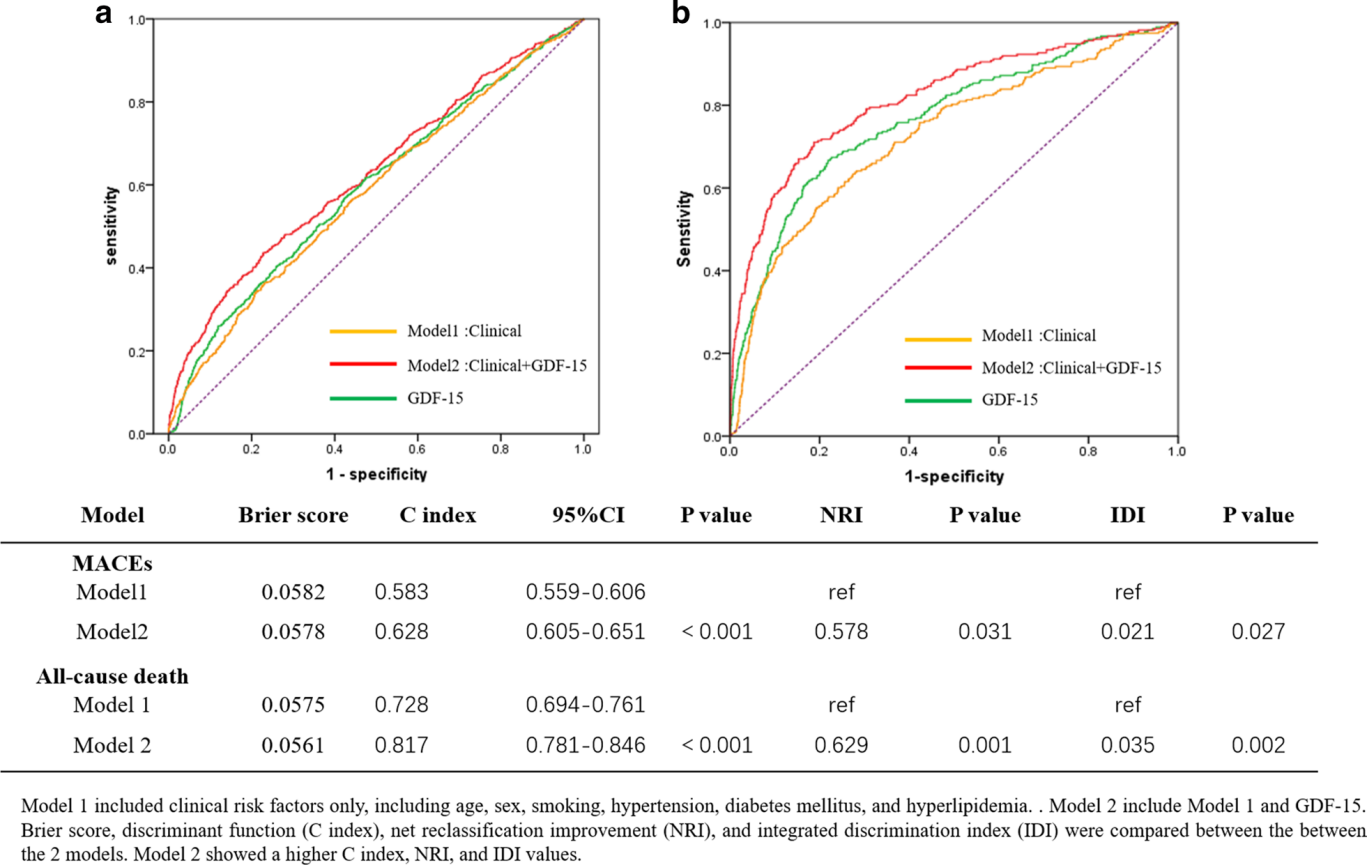
Comparison of different predictive models for predicting MACEs (**a**) and all-cause death (**b**)

We analyzed the predictive value of GDF-15 by ROC curve. For MACEs: ROC curve analyses indicated that C index (area under the curve) was 0.583 (95% CI 0.559–0.606) for clinical model (model1), 0.595 (95% CI 0.594–0.641) for GDF-15 alone, 0.628 (95% CI 0.605–0.651) for clinical model including GDF-15 (model2). ROC curve analysis showed non-significant differences in the clinical model alone compared with GDF-15 alone (P = 0.093), however, there was a significant difference compared to the clinical model with GDF-15 (P < 0.001, Fig. 3a). Furthermore, Model2 showed a significantly improved net reclassification improvement (0.578) and IDI (0.021), compared with model 1 (Fig. 3). For all-cause mortality: ROC curve analyses indicated that C index were 0.728 (95% CI 0.694–0.761) for clinical model (model1) 0.766 (95% CI 0.735–0.798) for GDF-15 alone, 0.817 (95% CI 0.787–0.846) for clinical model including GDF-15. ROC curve analysis showed significant differences in the clinical model alone compared to the clinical model with GDF-15 alone (P < 0.001), and there was a significant difference compared to the clinical model with GDF-15 (P < 0.001, Fig. 3b). Moreover, Model2 showed a significantly improved net reclassification improvement (0.629) and IDI (0.035), compared with model 1 (Fig. 3).

### Prognostic value of GDF-15 in long-term and short-term

**Fig. 4:**
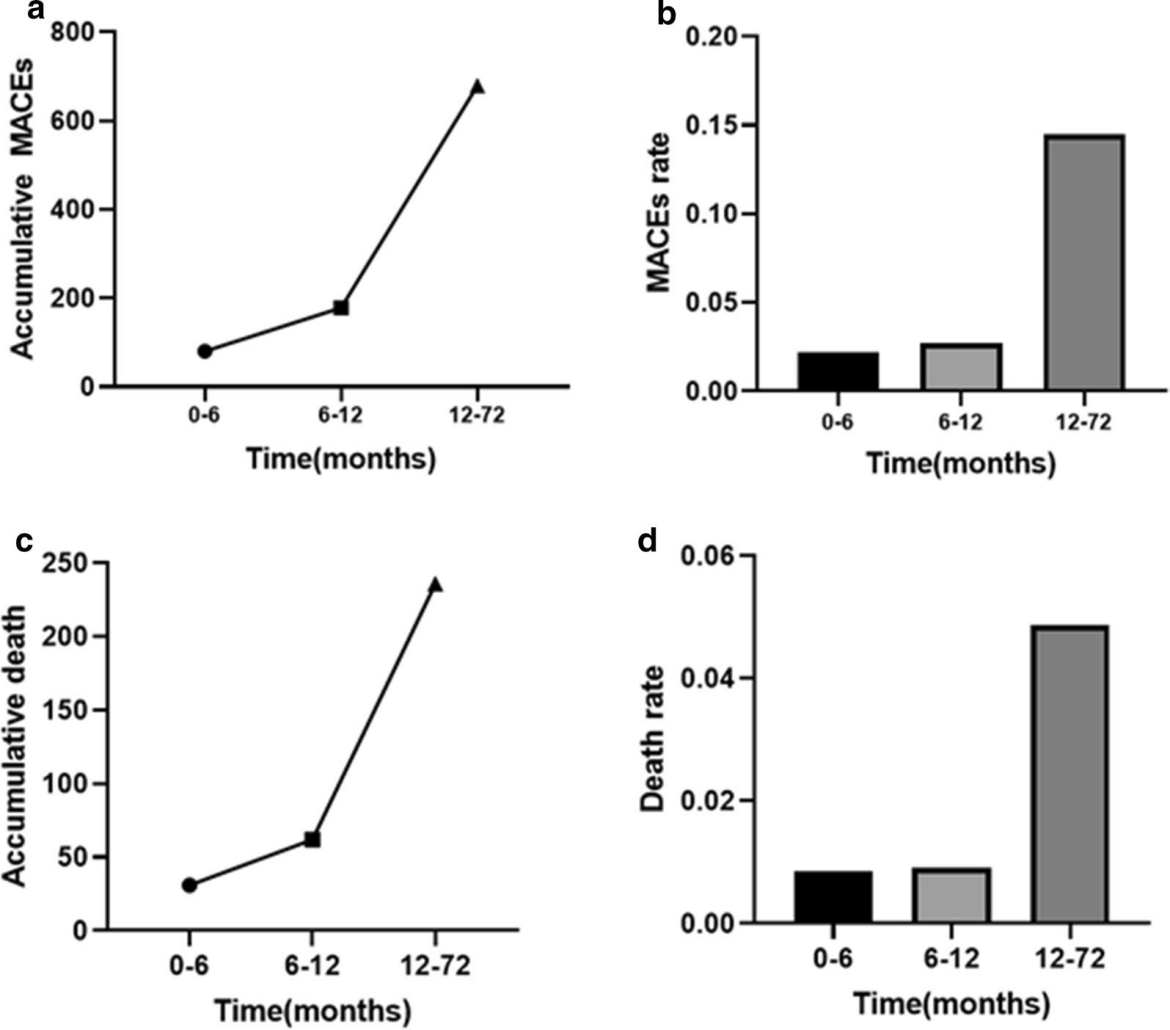
Accumulative MACEs (**a**) and death events (**c**) on 6 months, 12 months and 72 months, the MACEs (**b**) and death rates (**d**) during 0–6 months, 6–12 months and 12–72 months

**Fig. 5:**
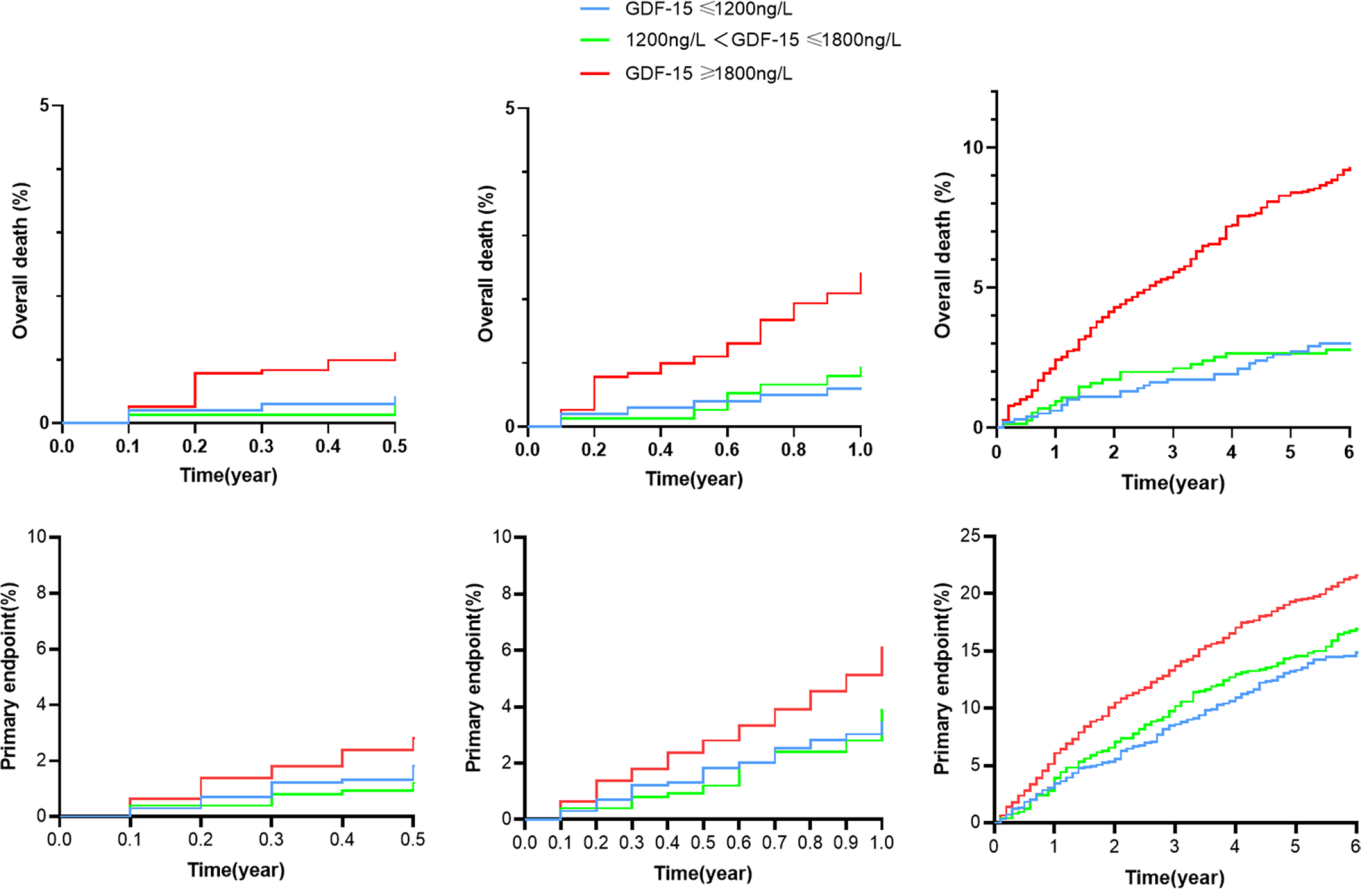
Kaplan-Meier analysis on 6 months, 12 months and 72 months according to different levels of GDF-15

During the follow up of 0–6 months, 80 patients had the occurrence of MACEs and 31 patients died. During the follow up of 6–12 months, 98 patients had the occurrence of MACEs and 33 patients died. During the follow up of 12–72 months, 501 patients had the occurrence of MACEs and 174 patients died (Fig. 4a, c). And the MACEs rates in 0–6 months, 6–12 months, 12–72 months were 2.20%, 2.68%, 14.47% respectively (Fig. 4b). All-cause death rates in 0–6 months, 6–12 months, 12–72 months were 0.85%, 0.91%, 4.86% (Fig. 4d). To evaluate the prognostic value of GDF-15, we also performed the Kaplan–Meier analysis upon different GDF-15 levels. The results indicated that the patients with GDF-15 > 1800 ng/L were significantly associated with an increased risk of all-cause death (log rank P = 0.021) in 0–6 months. But for MACEs, no significant difference was seen in 0–6 months (log rank P = 0.067). However, by the time of 12 months, the patients with GDF-15 > 1800 ng/L were significantly associated with an increased risk of all-cause death (log rank P < 0.001) and MACEs (log rank P < 0.001), the results were the same when they were followed up for 6 years (Fig. 5).

## Discussion

**Fig. 6:**
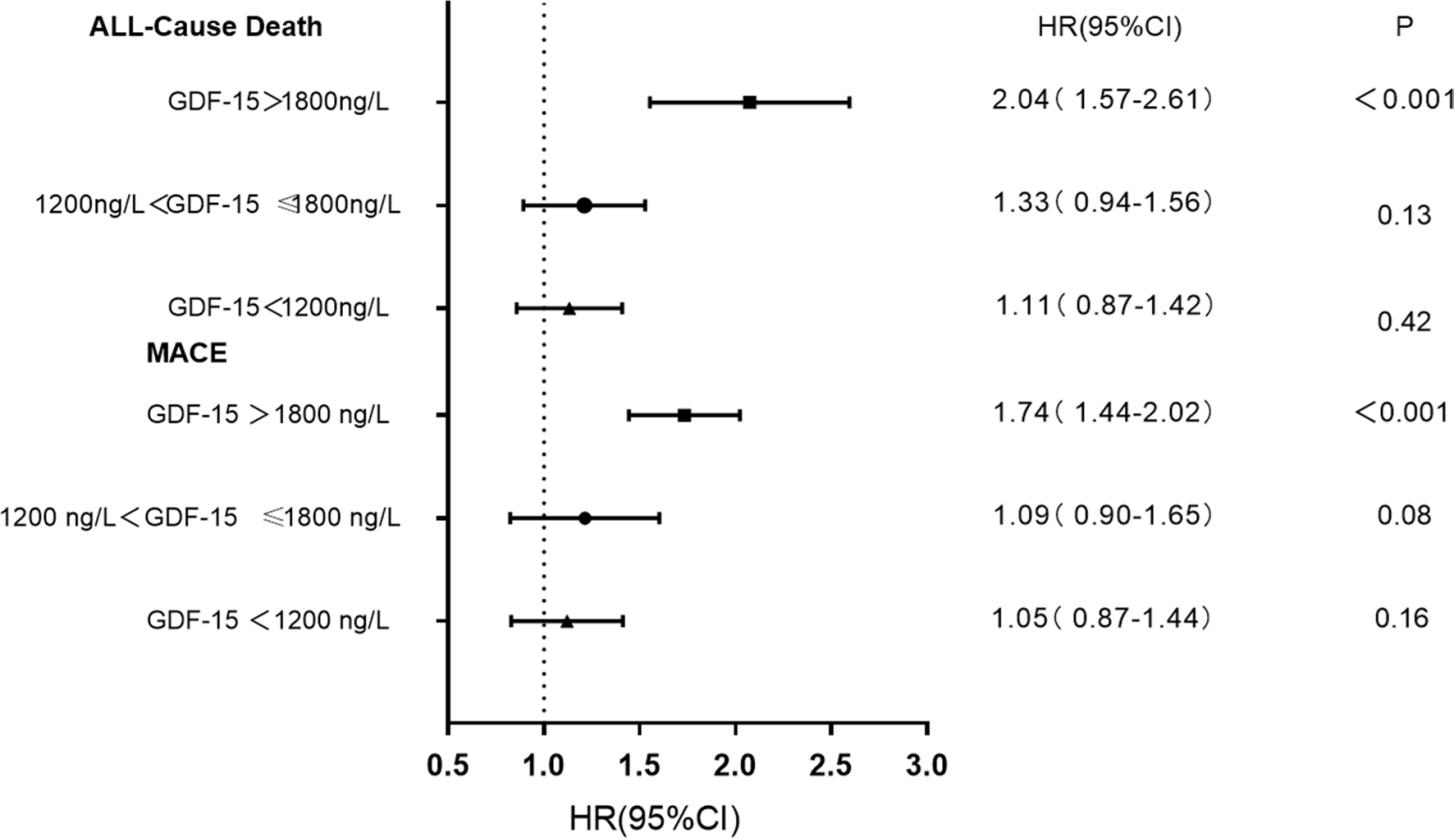
Hazard ratios, 95% confidence intervals (CI) according to values of GDF-15

In this study, we found that GDF-15 concentrations higher than 1800 ng/L were associated with an increased risk of all-cause death and MACEs in patients with established CAD. After adjusting for both established risk factors for CV disease and these other prognostic biomarkers, GDF-15 remained an independent indicator of MACEs and all-cause death (Fig. 6). Even more, we observed that GDF-15 provided an incremental prognostic value beyond a clinical model for MACEs and all-cause death. Besides, our research added new evidence for the short-term predictive value of GDF-15 for CAD patients. Finally, higher GDF-15 concentrations in the setting of established CAD were consistently related with an increased prevalence of cardiovascular risk factors, the result is in consistent with previous studies [16]. Our results provide updated information on the short-term and long-term prognostic role of GDF-15 in CAD, our result indicates that the addition of plasma GDF-15 measurements to information from clinical characteristics and established CV risk factors might further improve risk stratification.

### Inflammation and cardiac outcomes

Inflammatory processes are thought to actively trigger the development of CV disease and eventual clinical events. Many inflammatory cells such as neutrophils and macrophages might strongly influence atherosclerotic plaque stability, subsequently trigger acute thrombotic vascular disease, including myocardial infarction, stroke, and sudden cardiac death [17–19]. Many pro-inflammatory factors may influence cardiac remodeling as well as development of heart failure [20]. For instance, Cyr61 levels lead to augmented troponin and lower ejection fraction, and was independently associated with adverse cardiac outcomes [21]. Monocytes cells have been extensively proven to predict the incidence of myocardial infarction, the risk of MACEs also negative post-ischemic ventricular remodeling [22]. Moreover, inflammation is involved in cardiac muscle damage, neutrophils drive the early inflammatory response following a myocardial damage [23] and pro-inflammatory interleukins IL-6, IL-18 and MMP-12 are markers of preclinical cardiovascular organ damage [24]. Therefore, inflammation is involved in atherosclerotic plaque rupture, myocardial remodeling, myocardial injury, and other cardiac pathological processes, thus playing an important role in the outcome of cardiovascular disease.

### GDF-15 and potential mechanisms

Growth differentiation factor-15 (GDF-15), previous known as NSAID-activated gene 1 (NAG-1) and MIC-1, is a divergent TGF-β family member historically associated with cardiovascular disease and a host of other diseases with inflammatory etiologies [6, 25]. It has been reported as an inflammation-induced central mediator of tissue tolerance and it increases during tissue injury and inflammatory states [6]. A large number of studies have shown that GDF-15 increases in response to various stressors including reactive oxygen species and pro-inflammatory cytokines [26–28]. Besides, GDF-15 is highly expressed in response to different kinds of cytokines and growth factors like interleukin-1 (IL-1), tumor necrosis factor- (TNF-), angiotensin II, macrophage colony stimulating factor (M-CSF), and TGF- [29–31]. Tumor suppressor protein p53 also induces GDF-15 and thus acting as a growth inhibitory molecule in tissue [32]. Moreover, it has been reported that GDF-15 induced pro-inflammatory factors such as IL-1b, tumor necrosis factor-α, and CRP induce GDF-15 expression in macrophage cells through the regulation of p53 binding sites in the GDF-15 promoter [33]. The above studies have shown that GDF-15 is induced in inflammatory conditions, and GDF-15 is necessary for survival in inflammation conditions [6]. Therefore, we think GDF-15 might promote tissue protection from inflammatory damage thus playing an important role in the cardiac protection. But beyond that, a growing body of evidence supports that low circulating testosterone are correlated with adverse cardiovascular outcomes, including a higher incidence of CAD and increased cardiovascular and all-cause mortality [34]. It has been reported that in male patients with CAD, high GDF-15 levels are associated with testosterone deficiency supporting the idea that upregulation of GDF-15 in the presence of low testosterone is a potential mechanism by which GDF-15 affects CAD [35, 36]. Therefore, whether GDF-15 plays a role in cardiovascular protection by affecting testosterone levels or mainly through inflammatory response pathways, its intrinsic mechanism needs to be further elucidated.

### GDF-15 and cardiac outcomes

The prognostic value of GDF-15 has been reported for various cardiovascular diseases, such as ACS, atrial fibrillation, and heart failure [12, 14, 15, 37, 38]. Lindholm had reported a study included 17 095 patients with acute coronary syndrome, GDF-15 was the strongest marker associated with all-cause death [14]. According to Kempf T’s research, GDF-15 provided prognostic information in STEMI [12]. The predictive value of GDF-15 in ACS has been confirmed in the 2 large non-ST-segment-elevation ACS (NSTE-ACS) trials: the GUSTO-IV (Global Utilization of Strategies to Open Occluded Arteries IV) and FRISC II (Fast Revascularization during Instability in Coronary Artery Disease II) cohorts [12, 15]. Sharma A reported in the ARISTOTLE trial which included 18 201 patients with atrial fibrillation that GDF-15 was the strongest marker associated with bleeding death [37]. According to Bouabdallaoui N ‘s research baseline GDF-15 and changes of GDF-15 at both 1 month and 8 months were associated with subsequent mortality and CV events in patients with heart failure in the PARADIGM-HF trial [38]. GDF15 could be an integrative biomarker of heart failure in patient with AMI [39]. Moreover, in patients with ambulatory heart failure and reduced ejection fraction, GDF-15 is strongly associated with mortality and adverse cardiovascular outcomes [38]. Through the above researches, GDF-15 appears to be a promising biomarker for individual CV risk stratification. A lot of researches indicated that, GDF-15 together with a variety of markers may have a higher diagnostic value [12, 40, 41]. GDF-15 should probably be considered as a component of a multiple biomarkers’ cardiovascular score for future implementation in clinical practice [42].

According to previous researches, patients were divided into three groups: < 1200, 1200–1800, and > 1800 ng/L [12, 15], our research also divided our patients into three groups according to the GDF-15 levels, however, our result revealed that only GDF-15 values > 1800 ng/L were significantly associated with the incidence of MACEs in CAD patients. A research measured plasma GDF-15 in 3219 participants of the Dallas Heart Study, their result showed that GDF-15 > 1800 ng/L was associated with all-cause mortality (HR 3.5; 95% CI 2.1–5.9, P < 0.0001), and cardiovascular mortality (HR 2.5; 95% CI 1.1–5.8, P = 0.03).Our study is consistent with the results of this study [43]. We add new evidence that GDF-15 > 1800 ng/L maybe a high risk critical range for patients with coronary heart disease. According to our research, GDF-15 levels are predictive of both long-term MACEs and all-cause death, while GDF-15 can only predict all-cause death in the short-term. Previous studies have shown that GDF-15 has a short-term predictive value for ACS patients and a long-term prognostic value in stable CAD patients. Increased levels of GDF-15 were associated with a higher risk of death during 1-year follow-up in 741 STEMI patients [12]. Another study recruited 1142 patients with NSTEMI or STEMI were follow-up for 1.4 years, the result indicated that GDF-15 is a new marker for predicting death and heart failure in post-AMI patients [44]. Bonaca et al. reported 3501 patients with NSTE-ACS or STEMI were followed up 2 years, GDF-15 is associated with recurrent events after ACS [45]. A total of 1352 patients with stable angina pectoris were followed 3.6 years [46]. Hagström et al. reported that GDF-15 was independently associated with mortality in 14 577 patients with stable CAD during a follow up of 3.7 years [47]. According to the above studies, GDF-15 has a short-term predictive value for ACS patients and a long-term prognostic value in stable CAD patients. While the result of our research shows that GDF-15 levels are predictive of both long-term MACEs and all-cause death, and GDF-15 can only predict all-cause death in the short-term. Our results also add new evidence for the short-term predictive value of GDF-15 for CAD patients.

In conclusion, our research reveals the pathophysiological pathways of GDF-15 underlying CAD. Higher level of GDF-15 can predict the MACEs events and mortality for CAD patients, GDF-15 values > 1800 ng/L may be a critical value with a strong prognostic value. Proper reference ranges of GDF-15 need to be established to identify the disease severity and risk stratification of the diseases. Our study provides evidence for the high risk values range and add new evidence for the short-term predictive value of GDF-15 for CAD patients. But whether GDF-15 plays a role in cardiovascular protection by affecting testosterone levels or mainly through inflammatory response pathways, its intrinsic mechanism needs to be further elucidated. GDF-15 appears to be a promising biomarker for individual cardiovascular risk stratification, but a combination with other biomarkers may have higher predictive value for cardiovascular disease.

## Conclusions

In conclusion, this large sample size and long-term follow-up study indicated that in the setting of CAD, GDF-15 is associated with long-term all-cause death, MACEs and provides incremental prognostic value beyond traditional risks factors.

## Data Availability

The datasets used and/or analyzed during the current study are available from the corresponding author on reasonable request.

## Abbreviations

GDF-15: Growth differentiation factor-15
CAD: Coronary artery disease
MACEs: Major adverse cardiovascular events
CV: Cardiovascular
MI: Myocardial infarction
AMI: Acute myocardial infarction
ACS: Acute coronary syndrome
STEMI: ST-segment elevation myocardial infarction
M-CSF: Macrophage colony stimulating factor
TGF-β: Transforming growth factor-β
IL-1: Interleukin-1
TNF-: Tumor necrosis factor-
MIC-1: Macrophage inhibitory cytokine 1
HR: Hazard ratios
ROC: Receiver operator characteristic
PCI: Percutaneous coronary intervention
CABG: Coronary artery bypass grafting
TGF-b: Transforming growth factor b
TC: Total cholesterol
TG: Triglyceride
HDL-C: High density lipoprotein cholesterol
LDL-C: Low density lipoprotein cholesterol
IDI: Integrated discrimination improvement
NRI: Net re-classification improvement
